# Older Ghanaian adults’ perceptions of physical activity: an exploratory, mixed methods study

**DOI:** 10.1186/s12877-019-1095-1

**Published:** 2019-03-15

**Authors:** Laura E. Balis, Godfred Sowatey, Kwame Ansong-Gyimah, Eunice Ofori, Samantha M. Harden

**Affiliations:** 1 University of Wyoming, Laramie, Wyoming USA; 2 Koforidua Senior High Technical School, Koforidua, Ghana; 3 University of Education, Winneba, Ghana; 4 Virginia Tech, Blacksburg, Virginia USA

**Keywords:** Physical activity, Ghana, Older adult

## Abstract

**Background:**

Ghana is experiencing an epidemiological shift in public health issues toward non-communicable diseases that are underpinned by modifiable health behaviors. Physical activity rates have decreased, especially among older adults, coinciding with urbanization and an increase in sedentary work. Community-based physical activity programs are a recommended method of increasing physical activity levels; however, none currently exist in Ghana. The aim of this exploratory study was to determine older Ghanaian adults’ perceptions of physical activity and asses fit and feasibility of adapting and delivering a physical activity program for this population.

**Methods:**

Through a concurrent exploratory mixed-methods design, data were gathered from Ghanaian older adults (*N* = 123) during focus groups (*N* = 10) conducted at one diabetes clinic and nine churches across three urban areas. Qualitative data were collected using a semi-structured script that prompted for responses related to physical activity perceptions and the fit and feasibility of physical activity program characteristics. Quantitative data were collected through a questionnaire assessing participant demographics, physical activity levels, and health rating as well as physical activity knowledge and self-efficacy.

**Results:**

Findings indicate that older adults in Ghana have a need for and an interest in physical activity promotion. Participants had positive perceptions of being physically active, but were unaware of physical activity guidelines and how to meet them. Peer influence and health care providers’ recommendations were motivating factors for physical activity participation. As for desired physical activity program characteristics, participants expressed interest in group-based activities and becoming peer leaders and preference for a church-based program.

**Conclusions:**

The results suggest that a group-based physical activity program encouraged by health care providers and delivered at churches through a train-the-trainer model would be well received by aging adults from three urban areas of Ghana. In addition, education on physical activity types is needed, along with better dissemination and education on Ministry of Health physical activity guidelines. This exploratory work highlights preliminary support for a group- and community-based physical activity program for the aging population in Ghana. Beginning with the end in mind, these program characteristics should be considered when adopting, adapting, and implementing an intervention with this population.

**Electronic supplementary material:**

The online version of this article (10.1186/s12877-019-1095-1) contains supplementary material, which is available to authorized users.

## Background

Like other developing countries, Ghana (located in Sub-Saharan Africa) is experiencing a public health shift with increased incidence of non-communicable disease such as stroke, heart disease, and obesity [1, 2]. Notably, this increase in non-communicable disease rates coincides with a decrease in physical activity rates [3]. Engaging in regular physical activity decreases the risk of these chronic diseases as well as type 2 diabetes, cancer, osteoarthritis, and osteoporosis [4–6]. Additionally, for those who have been diagnosed with a chronic disease, physical activity can decrease the risk of premature death [5]. For example, for those with cardiovascular disease, staying physically active decreases risk of future cardiac events [5]. Overall, regular physical activity slows the progression of chronic conditions and increases both overall life expectancy and active life expectancy (i.e., years of life free of significant disease or disability) [7].

The Ministry of Health in Ghana recommends that older adults engage in 150 min of moderate-intensity aerobic activity (or 75 min of vigorous-intensity aerobic activity, or an equivalent combination of both) and two sessions of muscle-strengthening activities (targeting major muscle groups) per week [2]. However, physical activity participation has decreased with urbanization and the increase in sedentary jobs [3]. Physical inactivity rates are higher among women, those living in urban areas, and older adults [8]. Older adults (those aged 50 and older, per the World Health Organization’s definition of older adults in Africa [9]) are of particular concern as healthcare utilization to combat diabetes and obesity are also the rise [2].

Ghana is an ideal country in which to begin promoting physical activity in older adults to thwart the rise in non-communicable diseases. Ghana is a leader among African countries; it was the first sub-Saharan African country to achieve independence from a colonial power and is considered the “beacon of hope” of Africa due to its political stability, human rights, and economic growth [10, 11]. Addressing the new challenge of promoting physical activity in Ghana to halt the increases in chronic disease prevalence can set an example for other African countries to follow.

The public health concerns regarding the impact of urbanization, physical inactivity, and health disparities may be mitigated through community-based physical activity interventions. These interventions are an effective method of helping adults to begin and continue regular physical activity through using social networks to foster behavior change [12]. However, a systematic review of community-based older adult physical activity interventions did not identify any interventions in Ghana [13]. Community-based physical activity interventions been shown to be effective among diverse settings and populations [12]; they may also be effective in Ghana due to African cultural values that are centered on social relationships and support [14].

What is known about this population with regard to physical activity promotion comes from formative work conducted by Tuakli-Wosornu and colleagues [15]. This study explored physical activity and activity preferences of Ghanaian adult women, fitness professionals, and clergy through focus groups (*N* = 3) and in-depth interventions (*N* = 6 participants) with follow-up surveys [15]. Results indicated that female participants were primarily motivated to engage in physical activity by weight loss, health concerns, and “energy increases [15].” The participants’ main barriers to physical activity were time, work/family, and no available facility [15]. Walking, dancing, and group classes at the gym were the preferred types of physical activity [15]. Participants also stated a preference for group-based activities, as the social aspect is important to them [15]. It was also found that women often did not feel comfortable exercising at the gym [15]. Finally, the study found that clergy who participated in focus groups and interviews were interested in faith-based fitness programming [15]. While participants expressed an awareness of physical activity benefits and an interest in programming, they also reported uncertainty of what types of physical activity to recommend [15]. Although this preliminary work highlights some key considerations for intervention with Ghanaians, the sample was limited to women, few participants (< 50%) were 50 years of age or older, and only one setting was targeted (a church in one city) [15]. Therefore, to increase transferability and generalizability, work is needed to capture perceptions of aging males and females who live in various cities of Ghana.

Capturing perceptions of a wider target audience may improve the degree to which an intervention can be scaled within and outside of Ghana. In order to speed the translation of evidence-based programming into practice settings, researchers and practitioners should “begin with the end in mind [16].” That is, how an intervention will fit within a specific culture and setting, or be scaled [17] beyond its initial efficacy and feasibility testing, is often an afterthought of intervention development [18]. This is problematic because although the intervention may have internal validity (effective under tightly controlled conditions), it may not have external validity (generalizable to real-world settings) [16].

In addition, interventions that are developed and implemented without community-level decision-making may be a poor fit between the intervention and the setting [18]. Including local knowledge and considering local resources will improve the likelihood of a good fit [18]. Using a research-practice partnership can improve the translation of research to practice through collaborating with partner organizations to develop and deliver the intervention [19].

One system that is predicated on research-practice partnership principles is the U.S. land grant university Cooperative Extension System (herein: Extension). Community-based staff of Extension are uniquely positioned to pilot test an older adult physical activity intervention in Ghana because the United States Department of Agricultures’ National Institute of Food and Agriculture (NIFA), which funds Extension [20], created an initiative in 2003 to encourage the internationalization of Extension [21]. The goal of the initiative is to assist state Extension programs with adapting to today’s interconnected world and providing leadership in a global society [21]; however, these efforts have traditionally focused on agriculture, food security, and community development [22].

Therefore, the aim of this study was to determine older Ghanaian adults’ perceptions of physical activity and the characteristics of a physical activity program that would be acceptable to the population [23] that may be integrated into initiatives between Cooperative Extension and international audiences. The results of the study will be used to a) select an evidence-based intervention that can be adapted for this population and setting, and b) implement and evaluate the resultant program in Ghana.

## Methods

### Study design

The study employed a concurrent mixed-methods design [24] implemented through a collaboration with the second author, a physical education teacher who served as a research assistant. Qualitative data were gathered from Ghanaian older adults (potential program participants) (*N* = 123) through focus groups (*N* = 10) to determine their perceptions of physical activity and assess acceptability of the potential intervention, including desired instructors and locations. Quantitative data were gathered through a questionnaire administered to focus group participants (*N* = 121). The University of Wyoming Institutional Review Board approved this study (protocol #20170328LB01536).

### Recruitment

Focus group participants were recruited through adult church groups in three urban areas: Accra, Cape Coast, and Koforidua. These cities represent three of the twelve largest urban areas in Ghana and each represent a different region of the country (Greater Accra, Central, and Eastern). Churches in each area were sent a letter by the second author inviting them to participate; the diabetes clinic was invited through a conversation with a nurse practitioner. Participating churches and the diabetes clinic were then contacted to schedule focus groups and recruit participants. Church leaders and diabetes clinic staff used word of mouth and announcements to recruit individuals to attend the scheduled focus groups.

### Data collection

#### Qualitative

Focus groups were conducted at: Presbyterian Church of Ghana - Calvary Congregation in Koforidua (*N* = 3), Central Assemblies of God in Koforidua (*N* = 2), Resurrection Methodist Church in Accra (*N* = 1), and Ebenezer Methodist Church in Cape Coast (*N* = 2). In addition to the churches, focus groups were also held at the Diabetes and Hypertension Clinic in Koforidua (*N* = 2). Focus groups included nine to fourteen participants, except for the groups at Church of Christ, which included 19 participants each. Focus groups were larger than the recommended six to ten participants [25, 26]. This was due to overwhelming interest from the adult church groups as the focus groups were considered a “program” that all church group members typically attend. Focus groups held at the clinic were also large, as all clients attending the clinic that day were interested in participating.

Focus groups were moderated by the second author with the first author serving as an assistant moderator. Community volunteers (e.g., teachers and nurses) also provided assistance during the focus groups, especially by aiding participants with low literacy levels in completing consent forms and questionnaires. At three locations, two focus groups were completed simultaneously to accommodate large numbers of interested participants. In these cases, the second author moderated one focus group and a community volunteer moderated the other with first author as the assistant moderator.

Focus groups lasted two to two and a half hours due to the large sizes of the groups and the time necessary to read the consent form, questionnaire, and focus group questions in both English and Twi. Focus group participants were compensated for their time with 20 Ghana cedis (approximately $5). Community volunteers were also compensated with 20 Ghana cedis. The main contact at each church and at the clinic who coordinated the focus group(s) was given a gift (water bottle or cutting board) chosen by the second author to thank them for their assistance.

Focus group planning and script development were completed in partnership with the second author. Part of the semi-structured interview guide was developed based on the Theory of Planned Behavior [27] in order to explore attitudes, subjective norms, perceived behavioral controls, and intentions related to physical activity. The Theory of Planned Behavior is a health behavior theory that has been used to explain physical activity behaviors [28, 29] and can be used to develop, implement, and evaluate interventions based on the identified determinants of these behaviors [27]. Strength training perceptions were included in order to fully assess perceptions of the Ministry of Health’s recommendations [2].

Focus group questions also included exploring characteristics of a physical activity program that would be acceptable to Ghanaian older adults in order to ensure the intervention will meet the needs of the population and be well received [26]. Questions included the target participants and delivery agents, program location and schedule, delivery methods, and program contents and characteristics. Focus group participants were also asked about the feasibility of delivering a physical activity intervention themselves after going through the program to explore a potential train-the-trainer model [30]. Group dynamics [31, 32] items were included, as focus group results from Tuakli-Wosornu and colleagues’ study identified a preference for group-based activities among Ghanaian adult women [15]. Evidence shows that these group dynamics-based interventions have successfully increased physical activity levels [33]; however, many of these studies have been conducted in Western cultures, and understanding the preference for group-based or individual activities in other cultural settings is a necessary next step. Finally, intervention acceptability was also included in order to inform future dissemination and implementation. (See Additional file 1: Appendix A.)

#### Quantitative

Focus group participants completed a questionnaire on demographic variables, physical activity levels, health rating, physical activity self-efficacy, and knowledge of physical activity guidelines. Demographic variables included age, sex, ethnic group, education level, employment status, and marital status. Self-reported weight classification was used rather than self-reported height and weight to calculate BMI, as Tuakli-Wosornu and colleagues’ study found self-reported weight in this population to be unreliable [15]. Questionnaire items also included how long it took participants to get to the focus group location, how they traveled to the location, and where they live. The location was either their church or diabetes clinic.

The Global Physical Activity Questionnaire was used to determine physical activity levels over the last week [34]. The GPAQ is a valid and reliable instrument that has been tested in nine diverse countries. It is correlated with the International Physical Activity Questionnaire (IPAQ) but has fewer items, includes adults up to 75 years old (rather than up to 65 years old) [34, 35], and was also used in the World Health Organizations’ Study on global AGEing and adult health (SAGE) that included data on Ghana [3]. The GPAQ is also appropriate for this population as it includes work and travel physical activity [34]. (See Additional file 2: Appendix B.)

Participants were asked to rate their health compared to others their age on a four-point forced-answer scale of “Extremely healthy” to “Very unhealthy,” including a “Don’t know” option. To assess self-efficacy for physical activity, participants were asked how confident they are that they can engage in moderate physical activity for 30 min for five or more days a week using a five-point Likert scale from “Not at all” to “Completely” confident. They were asked to identify the physical activity recommendations for Ghanaian older adults and to identify the amount of physical activity they engage in compared to the recommendations (“Less than recommended amount,” “Meeting recommendations,” “More than the recommended amount,” “I do not engage in physical activity,” and “Unsure”).

### Analysis

#### Qualitative

Focus groups were audio-recorded. Notably, portions of the focus groups were conducted in Twi (Ghanaians’ native language). As suggested by Chen and Boore [36], the recordings were kept in original language for as long as possible. For this study, this meant that all recordings were transcribed in English by three of the authors, whereas the Twi portions were transcribed into English by the third and fourth authors, who are bilingual, using Microsoft Word. All coding was conducted by the first and senior author, who do not speak Twi. However, any cases of misunderstanding were checked with the Twi translators and the second author who conducted the focus groups. An inductive, grounded theory approach [37] was used to interpret the data. Transcripts were independently coded by the first and senior authors to identify meaning units (i.e., words, phrases, or sentences that contain related content that relays one specific thought or idea) [38]. After all meaning units were identified and categorized, the first author and senior author performed thematic sorting and collapsing of similar meaning units. Each coder reviewed the meaning units and subsequent analysis of the other coder. Frequencies of subthemes and categories were listed by both number of meaning units and number of focus groups to account for the large focus group sizes in which all participants may not have had a chance to speak. Audit trails [39] were maintained for all qualitative data, including focus group and interview audio recordings, transcripts, notes, and all coding documents.

#### Quantitative

Statistical analysis was conducted using SPSS. Means and standard deviations of continuous variables and frequencies and proportions of nominal variables were calculated for the overall sample. As the proportion of focus group participants was much smaller than the proportion of eligible older adults across the three cities in Ghana, descriptive statistics were used to describe focus group participants, but the degree of similarity between participants and the overall older adult population of Ghana was not calculated.

The Global Physical Activity Questionnaire (GPAC) items were used to compute Metabolic Equivalent Task (MET) values and calculate total physical activity. In accordance with GPAC data interpretation guidelines [40], MET values were calculate as a combination of the three domains (work, transport, and recreation). Values were then used to estimate mean physical activity through MET-minutes per week and classify the sample population as “active,” “insufficiently active,” or “inactive” according to Ghana Ministry of Health recommendations. GPAC items related to sedentary behavior were used to calculate median values of sedentary minutes per day. Participants with invalid responses to any of the domains or with no valid responses were removed from the data set (*N* = 60).

## Results

### Qualitative

The focus groups (*N* = 10) generated 1440 meaning units; they were coded into themes of perceptions of physical activity (*N* = 797 meaning units) and physical activity program characteristics (*N* = 643 meaning units). Meaning units around Perceptions of Physical Activity were divided into subthemes of A) preferred types of physical activity; B) barriers to physical activity; C) facilitators to physical activity; D) benefits of physical activity; E) factors that would encourage physical activity participation; F) current timing, location, and frequency of physical activity; G) physical activity recommendations; and H) potential negative consequences of physical activity.

Participants also expressed that they were active through their daily chores such that a participant shared: *“Even sweeping is exercise.”* Participants preferred dancing (*“I will put on music and dance [that will convince me to do aerobics]”)*, walking, and jumping (e.g., jumping rope) for aerobic activities. As for strength training, participants mentioned press-ups, weight lifting, and abdominal exercises.

Barriers to physical activity included lack of equipment and ill health, while facilitators included an individual’s strong determination and making physical activity a routine. Benefits of physical activity included strength and chronic disease prevention, such as heart health (*“[Physical activity] helps make the heart function well.”*) and weight management. Peer influence and health care providers’ recommendations were identified as factors that would encourage physical activity participation. Promoting the physical activity program on TV could also motivate participants to attend, as several participants mentioned televised calls to exercise or exercise programs that motivated them to be active: “*So, when you turn on the TV you see people exercising. And because they recommend that it is something that is good to do so everyone should do it. So, when you see that, it encourages you to also exercise.”*

Most participants preferred to exercise in the mornings inside their homes, with several respondents across multiple focus groups mentioning that they could exercise in their bedrooms. Participants expressed mainly positive perceptions of the Ministry of Health physical activity recommendations but also expressed concerns about their age appropriateness: *“If you are above 60, you will be exhausted, and that will be dangerous for you….”* Finally, participants also mentioned potential negative consequences of physical activity, including injury, soreness, and undesired weight loss.

Meaning units around the theme of Physical Activity Program Characteristics were divided into subthemes of A) meeting preferences; B) need for education; C) leader preferences; D) need for group-based program; E) delivery method; F) activities; G) incentives; and H) group similarity. Based on the participants’ meeting preferences, the program would likely take place at churches and meet once a week for one hour. Most participants preferred meeting on Saturdays due to work commitments during the week and church on Sundays, while activities that take place on Saturdays were also mentioned as a barrier: *“Most of activities occur in the weekend, I have to go to funeral, baby naming ceremony, weddings….”* Church leaders who participated in the focus groups supported the idea of hosting a physical activity program: *“…On behalf of the church, we want to say [participating in this focus group] has helped us to know that we have to exercise our bodies. And we promise as a church that we will form a Keep Fit club here. So that every weekend or once in a month, we will be meeting and then exercise our bodies.”*

Participants conveyed a need for nutrition education, including timing and type of food to eat when participating in a physical activity program as well as general nutrition. Several participants mentioned a need for a dietitian to provide education: “…*if we get somebody, let say a dietitian, who can be coaching us on our diet sometimes, by telling us… you need to eat this, you need to stop eating this, that will help.”* They also expressed a need for education on types of physical activity (e.g., what counts as aerobic exercise and strength training), and felt that the Ministry of Health should be involved in educating the public.

As for who should lead the program, participants desired someone with training in physical activity. However, when asked if they could lead the program after going through it, the participants agreed that they could become peer leaders. Most participants preferred a leader the same age as them *(“We want someone like us…our peer”)*, as well as someone who is knowledgeable, organized, and enjoyable. Participants also voiced a preference for a group-based activity program, mentioning several components of group cohesion, including group norms, distinctiveness, competition, and size [33] *(“And if we come and there are enough older people participating, it encourages others to come”)*. Overall, participants had positive perceptions of group-based programs and felt that this type of program would increase motivation.

As for delivery method, participants expressed a preference for an in-person physical activity program. Some participants had positive perceptions of other delivery methods (e.g., DVD, email, WhatsApp), but also mentioned that it is difficult to determine if information on WhatsApp or other social media comes from a trusted source. It was also noted that access to the delivery method needs to be considered, as internet access can be expensive and participants may not use social media: “*It is possible that someone may not have WhatsApp….”*

As for program activities, participants mentioned interest in active games (e.g., ampe which is a children’s jumping and clapping game), health screenings (e.g., blood pressure checks), and health talks (e.g., talks given by physicians). Refreshments were the preferred form of incentives for physical activity program participation, with participants mentioning water, soft drinks, fruit drinks, crackers, bread, fruit, and vegetables. Another desired incentive was financial support, especially to help with transportation. Some participants also suggested paying dues to support each other: “*Maybe you can have some dues that you pay, in case there is a problem with any of the members.”* Finally, as for group similarity, most participants preferred a mixed-gender program (Tables 1 and 2).

**Table 1:** Qualitative results for theme: perceptions of physical activity

Subtheme	Category	Example meaning unit
Preferred types of physical activity (*N* = 205 MU, *N* = 10 FG)	Daily chores (*N* = 36 MU, *N* = 10 FG)	Sweeping, sweeping in the morning, for exercise.
	Walking (*N* = 31 MU, *N* = 9 FG)	I always walk, walking everyday… [inaudible] wherever I’m going I walk.
	Dancing (N = 24 MU, N = 10 FG)	Dancing, using dance for exercises…when you dance to music... all of that is exercise.
	Jumping (*N* = 16 MU, N = 7 FG)	Using the skipping rope [is exercise]…
	Press-ups (*N* = 13 MU, N = 5 FG)	Yes, even press ups and … you can strengthen your muscles.
	Lifting weights (*N* = 11 MU, *N* = 4 FG)	And then weight lifting [I would do for exercise].
	Jogging (N = 9 MU, N = 6 FG)	The exercise we know in Ghana here is jogging.
	Team sports (N = 7 MU, N = 4 FG)	I do some small volley.
	Stretching (N = 6 MU, N = 3 FG)	I would also want to stretch my arms… stretching my neck.
	Abdominal exercises (N = 5 MU, N = 4 FG)	Twisting the waist [you can do for exercise].
	Other aerobic exercise (N = 4 MU, *N* = 4 FG)	Swimming too, swimming too…. He wants a swimming pool … we don’t know how to swim, we live here, but we don’t know how to swim.
	Other strength training (N = 4 MU, N = 4 FG)	I do a lot of muscle strengthening activities, but it’s not weightlifting.
	Tennis (N = 3 MU, N = 1 FG)	If you know how to play long tennis [for exercise].
Barriers to physical activity (*N* = 157 MU, N = 10 FG)	Lack of equipment (*N* = 48 MU, *N* = 10 FG)	The environment, here we don’t have the means, see the machines we don’t have them, so you wouldn’t know where [to exercise].
	Ill health (*N* = 25 MU, *N* = 9 FG)	Like maybe you are sick [so you cannot do physical activity].
	Laziness (N = 24 MU, N = 9 FG)	One big hindrance [to exercising] will be laziness.
	Lack of time (*N* = 19 MU, *N* = 8 FG)	In our setting, for instance, even though we are retired, there are other activities, sometimes you would be required to move, around 5:30, you are going to a family house somewhere, you are traveling, you are doing this, so many other activities, so actually, there would not be the time for you to be doing these things. Except on very few occasions, that you will have time for yourself.
	Poverty (N = 8 MU, N = 3 FG)	You are living in poverty (laughter). In poverty (laughter).
	Transportation (N = 8 MU, N = 4 FG)	Means of transport is also I mean very difficult when the exercise is far. Eh you have to get a means of transportation for the old people because not a lot of time they can come to do the exercise.
	Weather (N = 7 MU, N = 2 FG)	At times when it is rainy season, it makes it very difficult…
	Lack of appropriate programs (N = 4 MU, N = 3 FG)	[We need something specific for older adults] rather than neglect, total neglect.
	Tiredness (N = 4 MU, N = 3 FG)	But after a while me myself I will feel tired.
	Lack of interest (N = 4 MU, N = 3 FG)	Lack of interest, lack of interest [will prevent me from exercising].
	Safety concerns (N = 4 MU, N = 2 FG)	But if you have no other place but, but just walking by the roadside, you have heard several times that people go for walking, they’ve been knock down.
	Perception of strength training (N = 3 MU, *N* = 3 FG)	As for me, I don’t like muscle training…
Facilitators to physical activity (*N* = 94 MU, N = 10 FG)	Determination (*N* = 37 MU, N = 8 FG)	You have to be determine, it’s determination that we are talking about, determination, the will power, that is determination. If you have the determination, you have the will power to do it.
	Routine (*N* = 27 MU, N = 10 FG)	Well, I said that, you know, you’ve been doing this for a while, and you feel good when you do it, so skipping for a day will make you have a nagging feeling of missing something, because it has become part of you, and so you will …
	Music (N = 9 MU, N = 6 FG)	Music…. motives me… to exercise, yes, yes.
	Family support (N = 7 MU, N = 4 FG)	If you have children too, then you will let them be part of it. Sometimes they will even prompt you, let’s go and do this
	Healthy diet (N = 6 MU, N = 2 FG)	Then, uh, you know, diet has a lot in influence on our body.
	Keep Fit club (N = 5 MU, N = 4 FG)	So please, know that we have just formed a Keep Fit club in the church. We will meet at least once a month.
	Good health (N = 3 MU, N = 2 FG)	When you are healthy [you can meet physical activity recommendations].
Benefits of physical activity (*N* = 90 MU, N = 10 FG)	Overall health (N = 16 MU, N = 8 FG)	You become healthy, healthier than normal [when you exercise].
	Strength (N = 15 MU, N = 7 FG)	For people who are not strong, it [being physically active] helps strengthen your muscles and makes you stronger.
	Heart health (N = 13 MU, N = 6 FG)	[Exercise] makes your heart good strong.
	Weight management (N = 9 MU, N = 4 FG)	And, [exercise] also helps to reduce weight too.
	Sweating (N = 7 MU, N = 6 FG)	Well, I think that exercising allows you to sweat.
	Fitness (N = 6 MU, N = 4 FG)	[And if you exercise] it helps you to get fit.
	Mental health (N = 6 MU, N = 2 FG)	There is another type of issue, generally as we interact during that, you know, period of exercising emotionally you realize that people get over uh... some of the emotional problems. Like if they are widows amongst us, if they are people who are being stressed out and all those kind of things, it will help.
	Long life (N = 6 MU, N = 3 FG)	We will be able to live long [if we exercise].
	Sleep (N = 4 MU, N = 2 FG)	You will be able to sleep [if you exercise].
	Diabetes management (N = 3 MU, *N* = 2 FG)	Your sugar level also, can can can come down [if you exercise].
	Osteoporosis prevention (N = 3 MU, *N* = 2 FG)	[Exercise] makes your bones strong.
	Joint health (N = 2 MU, *N* = 2 FG)	When you exercise, it strengthens your joints.
Factors that would encourage physical activity participation (*N* = 89 MU, *N* = 9 FG)	Peer influence (N = 25 MU, *N* = 8 FG)	What will convince me is when I see my age mates engage in these exercises without suffering any adverse effects, such as bodily pains. I believe that will convince me to exercise.
	Health care provider recommendation (N = 15 MU, N = 6 FG)	When I visit the hospital, the doctor can let me know that I need to exercise, so the doctor can convince me to exercise.
	Gradual progress (*N* = 14 MU, N = 7 FG)	And when you do it [exercise] and you feel it… that is going on well for you, you are motivated to do more.
	Physical appearance (N = 14 MU, N = 4 FG)	Okay…when you see how the body is like, it will convince you [to do muscle strengthening activities]…yeh.
	Focus group information (N = 11 MU, N = 6 FG)	What I want to say is that, in fact, on the whole the program has been educative and very err, errr, very successful. We want to, on behalf of the church, thank you and your colleagues for bringing this program to our doorstep, and we promise as a church, that we are going to start a Keep Fit club in the church, so that every weekend or once in a month we will meet as a church members, exercise our bodies, so that we can grow healthy, and then live long.
	TV programs (N = 7 MU, N = 6 FG)	When you watch the TV, you can see people of the same age as you exercising and that person will be healthy and you sit down and ask yourself why are these people doing it and I am not?
	Existing knowledge (N = 3 MU, N = 2 FG)	I don’t think we need anybody to convince us. We know that it’s [exercise] necessary.
Current timing, location, and frequency of physical activity (N = 60 MU, N = 9 FG)	Time of day (*N* = 32 MU, *N* = 9 FG) Morning (*N* = 26) Evening (*N* = 5) Any time (*N* = 1)	You know, I think it is better in the morning, before you take your bath.
	Location (N = 23 MU, *N* = 7 FG) Home (*N* = 17) Gym (N = 2) Mountains (N = 2) Streets (N = 1) Church (N = 1)	At times, in my bedroom, or in the hall [I would do aerobic exercise].
	Frequency (*N* = 5 MU, N = 2 FG) Four days a week (*N* = 3) Three days a week (*N* = 1) Daily 1 (N = 1)	[I will do exercise] and will do it four days in the week.
Physical activity recommendations (*N* = 52 MU, N = 9 FG)	Positive perception (*N* = 23 MU, N = 7 FG)	It’s ok. If you are able to do at least 30 min even a day, iii is good it’s good for the body
	Concern about age appropriateness (*N* = 20 MU, N = 7 FG)	[I] feel it’s [the recommendations] a bit harsh. As elderly as I am, it’s harsh. If you are about 90 years.
	Neutral perception (*N* = 6 MU, *N* = 4 FG)	We, we are saying that [exercise] should be in moderation.
	Negative perception (N = 6 MU, N = 3 FG)	You cannot do it [meet physical activity recommendations].
Potential negative consequences of physical activity (*N* = 50 MU, *N* = 10 FG)	Injury (N = 16 MU, *N* = 8 FG)	It [exercise] may even cause them [your joints] to dislocate.
	Soreness (N = 14 MU, N = 7 FG)	Initially, you also feel the pains, the pains [if you exercise].
	Lose weight (N = 7 MU, N = 2 FG)	Your weight...that is if you do exercise you become [too] slim.
	Heart problems (N = 5 MU, N = 4 FG)	Palpitate… some people palpitate [during exercise]…
	Lack of energy (N = 5 MU, N = 4 FG)	It [physical activity] may not give you enough energy to work.
	Difficulty sleeping (*N* = 3 MU, N = 1 FG)	Please, I learnt that when you do it [exercise] in the evening, it makes your sleep to be very difficult… that’s what I learnt.

**Table 2:** Qualitative results for theme: physical activity program characteristics

Subtheme	Category	Example meaning unit
Meeting preferences (*N* = 142 MU, N = 10 FG)	Program location (*N* = 67 MU, *N* = 10 FG) Church (*N* = 27) Park (*N* = 12) Diabetes hospital (*N* = 6) Field (*N* = 4) Gym (N = 4) Conference room (N = 3) Spacious place (N = 2) Stadium (N = 2) Close to home (N = 3) Clubhouse (N = 1) Car park (N = 1) Community center (N = 1) Depends on program (N = 1)	If you are living around this place, perhaps you can use the Church [for an exercise program].
	Program frequency (*N* = 38 MU, *N* = 10 FG) Once a week (*N* = 24) Three times a week (*N* = 10) Twice a week (N = 6) Once a month (*N* = 4) Twice a month (N = 2) Daily (N = 1) Holidays (N = 1)	Once a week, you will got the office from Monday to Friday, on Sunday too, you must prepare for church, so Saturday is good for that.
	Program duration (N = 37 MU, *N* = 9 FG) One hour (*N* = 18 MU) Two hours (*N* = 10 MU) Thirty minutes (*N* = 5 MU) Ninety minutes (N = 4 MU)	One, one hour will be okay [for the class].
Need for education *N* = 110 MU N = 10 FG	Nutrition (*N* = 62 MU, N = 9 FG)	You will need to have someone teach us about what foods are good for us, and what foods are not. So if, we have someone who leads us in this direction, we will become more careful with our eating habits, even help with the supply of foods, such as the fruits that they talk about, that will help us.
	Types of physical activity (*N* = 23 MU, N = 9 FG)	She is asking if she can lie down and do [aerobic activity in bed].
	Ministry of Health involvement (*N* = 7 MU, N = 4 FG)	I also think that if it’s a recommendation, then they [Ministry of Health] should educate the public. Because this is the first time I’m hearing from you.
	Lack of information (N = 7 MU, N = 5FG)	I don’t get any health information from anybody…
	Benefits of physical activity (N = 5MU, *N* = 5 FG)	Also, I would like to be told what the negative consequences of not engaging in exercises may be.
	Injury / illness considerations (N = 4MU, N = 3 FG)	Okay, please, we mentioned several exercises that we could do, and my question is that for someone who may have a problem such as a bad knee and therefore cannot do certain exercises, are you able to provide any recommendations, as to what exercise may be appropriate?
	Unsafe recommendations (N = 2 MU, *N* = 2 FG)	We are also advice to walk barefooted but our foot can be pierced by broken glass or any harmful item. So in fact this sickness of ours [diabetes] has become a sickness you can’t really understand and we don’t know the where about of it.
Leader preferences *N* = 109 MU *N* = 10 FG	Qualifications (*N* = 65 MU, N = 10 FG) Trained instructor (*N* = 26 MU) Peer leader (*N* = 24 MU) PE teacher (*N* = 9 MU) Resource person (*N* = 4 MU) Health care provider (N = 2 MU)	Anybody who is well-versed in that act, in physical education can lead us. He would know what ages and what exercises to apply for us.
	Age preference (N = 24 MU, *N* = 8 FG) Same age (N = 10 MU) Younger (*N* = 6 MU) Any age (N = 8 MU)	One this is if the instructor is below 40 years of age, he will kill us [laughter] yes, it’s true [amid laughter] because the young can bend and they will be asking us to bend also you know [laughter].
	Traits (*N* = 16 MU, *N* = 5 FG) Knowledgeable (N = 5) Organized (*N* = 4) Enjoyable (*N* = 3) Strong (*N* = 2) Active (N = 1) Encouraging (N = 1)	Those who have the knowledge and skill in training people [should teach the class].
	Gender preference (N = 4 MU, *N* = 3 FG) Same gender (N = 2) Any gender (N = 2)	…but a man should train a man and a woman should train a woman.
Need for group- based program *N* = 72 MU *N* = 9 FG	Motivation (*N* = 19 MU, N = 7 FG)	It [group physical activity] encourages you to do more.
	Group cohesion (N = 13 MU, N = 4FG)	You have the same family, what concerns one also concerns you.
	Group norms (N = 11 MU, N = 6 FG)	…I would encourage the members to be punctual, to be punctual, I’ll encourage the members to be punctual.
	Positive perception (N = 10 MU, N = 6FG)	So if you have a group that you can engage in the exercise with, it helps a lot.
	Accountability (N = 8 MU, N = 5 FG)	The group is the best because, if you are lazy to go, they will come and call you hahaha yes. They will come to you, that one also.
	Enjoyment (N = 8 MU, N = 5 FG)	When we work together, it will help us, and we will be happy.
	Distinctiveness (N = 3 MU, N = 3 FG)	Please, I think things such as clothing and shoes that we will wear for the program, that identifies us to all that we are one group involved in exercise, will help.
Delivery method N = 62 MU N = 10 FG	In person (*N* = 36 MU, N = 9 FG)	So I wanted to say is that, (crosstalk) if the person is standing before you, you have an advantage and if he is explaining, that will help you to better understand what he is saying. But if the information is on CD, you can’t even ask questions. So if the person is before you, that’s the best.
	DVD (N = 6 MU, N = 5 FG)	DVD, because with that you can also see the [exercises demonstrated]...
	Electronic communication (N = 5 MU, N = 2 FG)	I will prefer information through email.
	Social media (N = 4 MU, *N* = 4 FG)	That is very good, it helps [to get information via WhatsApp].
	Need trusted sources (*N* = 3 MU, N=3 FG)	Know the person…qualification, who is talking to you… Facebook, err, online, on those things you don’t know, sometimes they are misleading.
	Consider cost (N = 3 MU, *N* = 3 FG)	It is useful but it is expensive, it is expensive, even even how to acquire all those things, even even you tube, you can’t just go there and be watching all these things… even the DVD, the internet work is expensive…but how to maintain them is expensive but it is good.
	Mass media (N = 3 MU, *N* = 3 FG)	People do listen to the radio, majority have radio.
	Individual program (N = 2 MU, N = 1FG)	Well, for me, I don’t believe that you have to join a group before you exercise. You should, yourself, engage in mini exercises. So in my own home, or I can walk. I can do it according to my own song. I don’t look at others to live my life. I do what suits me.
Activities N = 60 MU N = 9 FG	Active games (N = 13 MU, N = 7 FG)	Playing ampe [we would do as a group].
	Health screening (N = 11 MU, N = 7 FG)	And it would be advisable that when you are in a group, sometimes you have, you invite medical… err... doctors, to at least see if we fall in the range, that is they can take our BP they can advise what to do…
	Physical activity (N = 11 MU, N = 6FG)	[We would do] various exercises that will cater for the whole body.
	Health talk (N = 9 MU, N = 4 FG)	Sometimes, sometimes we have to invite a resource person, a resource person, to give us some talks. Someone like a doctor.
	Board games (N = 8 MU, N = 3 FG)	[We would play] ludo, and the draft.
	Group events (N = 4 MU, N = 3 FG)	At times we’ll also, eat, eating!
	Prayer (N = 2 MU, *N* = 2 FG)	And then, as Church members we pray, we pray. After the act...before the activity we pray. After the activity we say a word of prayer before we disperse…
	Singing (N = 2 MU, N = 2 FG)	We will sing, we learning how to sing.
Incentives *N* = 53 MU N = 10 FG	Refreshments (*N* = 30 MU, N = 9 FG) Prefer refreshments (*N* = 28 MU) Refreshments not needed (N = 2 MU)	Oh, maybe [we would have] a fruit juice [as a refreshment].
	Financial support (*N* = 15 MU, N = 7FG)	Maybe you can have some dues that you pay, in case there is a problem with any of the members
	Incentive items (*N* = 5 MU, N = 3 FG)	Motivation, like getting some t-shirt to wear.
	Transportation (N = 3 MU, N = 1 FG)	Transportation [will motivate people to get involved in the program].
Group similarity *N* = 35 MU N = 10 FG	Gender similarity (N = 30 MU, N = 9FG) Mixed-gender (*N* = 21 MU) Separate gender (N = 6 MU) Blended program (N = 3 MU)	Oh no, it doesn’t matter, when we are all together it’s okay! Men and women, it doesn’t matter at all… everybody can do it.
	Separate by age (*N* = 5 MU, *N* = 4FG)	Please, doing it in groups also helps… so like 70 going, their way of exercising, group groups, by age groups.

### Quantitative

One hundred twenty-one focus group participants completed questionnaires. These participants had a mean (±SD) age of 62.74 (± 7.74) years, were predominantly Akan (71%), married (71%), college educated (42%), and currently working (42%). See Table 3 for detailed demographic variables of the participants. As for self-reported weight status, 119 participants responded to this item, with 79% reporting normal weight, 13% overweight, 2% obese, 1% underweight, and 6% not sure.

**Table 3:** Demographic variables of focus group participants (*N* = 121)

Demographic variables	*N* (%)
Age	
50–54	22 (18)
55–59	25 (21)
60–64	27 (22)
65–69	21 (17)
70–74	15 (12)
75–80	11 (9)
Sex	
Male	52 (43)
Female	68 (56)
Not reported	1 (1)
Ethnic Group	
Akan	86 (71)
Ga-Adangbe	13 (11)
More than one ethnicity	9 (7)
Ewe	7 (6)
Guan	5 (4)
Other	1 (1)
Education level	
College	50 (41)
Secondary high school	36 (30)
High school	17 (14)
Primary school	14 (12)
No formal education	3 (2)
Not reported	1 (1)
Employment status	
Currently working	51 (42)
Retired	41 (34)
Not currently working	22 (18)
A homemaker	6 (4)
Disabled/unable to work	1 (1)
Never worked	0 (0)
Marital status	
Married	86 (71)
Widowed	20 (17)
Divorced	8 (7)
Single	4 (3)
Living with partner	2 (2)
Separated	1 (1)

Of the 117 participants who responded to the item related to health status, 48% percent of participants self-reported that they were somewhat healthy compared to others their age. Forty two percent reported that they were extremely healthy, 9 % reported that they were not healthy or very unhealthy, and 2 % did not know. Of the 120 participants who responded to the item on identifying physical activity recommendations, 43% were able to correctly identify recommendations, 53% were incorrect, and 4% were unsure. Of the 118 participants who responded to the item on meeting physical activity recommendations, 51% reported that they are meeting recommendations, 28% reported that they get less than the recommended amount of physical activity, 9% reported that they get more than the recommended amount, 6% reported that they do not engage in physical activity, and 6% were unsure.

Twenty eight percent of participants were very or completely confident that they could engage in this amount of activity (“moderate physical activities (e.g., not exhausting, light perspiration) for 30 minutes for 5 or more days per week”). Eight percent were not at all confident, 17% were somewhat confident, and 47% were moderately confident.

Thirty seven percent of participants reported that it took then 11–20 min to travel to church. Thirty two percent took 10 min or less to get to church, 16% took 21–40 min, and 14% took over 40 min. Fifty six percent of participants traveled to church by car, 38% walked, 3% took the bus, and 3% traveled to church by car and walking. Seventy six percent of participants live in Koforidua or the surrounding area, 16% live in Cape Coast, 7% live in Accra, and 1% live in “other” (not specified).

Out of 61 valid responses to the GPAQ questionnaire items, 82% of participants reported that they are meeting physical activity recommendations (greater than or equal to 600 METs), while 3% reported being insufficiently active (less than 600 METs) and 16% reported no physical activity. Mean (±SD) physical activity level was 7673 (± 10,307) METs (equivalent to over 31 h of moderate intensity or over 15 h of vigorous intensity physical activity per week). Mean (±SD) sedentary time was 233.9 (± 164.4) minutes per day.

## Discussion

This purpose of this exploratory study was to inform the implementation of an older adult physical activity program in Ghana. This information was sought to address an emerging need for physical activity promotion and chronic disease prevention for the aging population of Ghana. The qualitative and quantitative results of the study provide insight into older Ghanaian adults’ self-reported health and physical activity status, perceptions of physical activity, and desired physical activity program characteristics. Overall, the results suggest both a need for physical activity programming and interest in a community-based program. These results can inform the selection, adaptation, and delivery of an evidence-based older adult physical activity program by illuminating intervention characteristics that may be a good fit within the intended audience. Considering the perceptions of end users from the onset may speed the translation of programs from research to sustained practice [16].

The quantitative results showed that less than half of participants could correctly identify Ministry of Health physical activity recommendations. This was also expressed during the focus groups. Determining participants’ physical activity levels was difficult, as there was a discrepancy between the percentage of participants who self-reported meeting physical activity guidelines (51%) and the percent who reported through the GPAQ questionnaire that they meet guidelines (82%). Only 61 participants (50% of those completing questionnaires) had valid responses to the GPAQ items, and those who responded that they meet guidelines indicated that they accumulated much more than the recommended amount (over 31 h of moderate intensity physical activity per week compared to the recommended 2.5 h). Therefore, these values were likely over-reported; this may be due to a lack of familiarity with types of physical activity and intensity levels.

Indeed, a lack of knowledge of physical activity types emerged as a subtheme. Participants were unfamiliar overall with what counted as physical activity *(“So, is dancing a form of exercise?”* and “*I’ll clap my hands vigorously… that is part of it…clapping for long periods of time, it’s part of exercising…”),* types of aerobic activity *(“Stretching [is aerobic]”)*, and types of strength training *(“I’ll go jogging…, running, and just swinging my arms [for muscle strengthening]…”).* Daily chores were also mentioned as a type of physical activity participants engage in; this activity would likely be considered light rather than moderate or vigorous intensity. These results suggest respondents may *not* be meeting physical activity recommendations, and that there is a need for physical activity promotion and education. The results also suggest that to better understand current physical levels among older adults in Ghana, objective measures may be necessary. It is recommended that future studies, if funded, objectively assess physical activity levels through culturally appropriate measures.

In addition, participants felt that the Ministry of Health should be more involved in promoting physical activity: *“…this will come from Ministry of Health. They have to send someone to educate you, instructors, to encourage you by instructing you do this, do like this.”* A Ministry of Health educational campaign complementing the physical activity program could increase the number of older adults who are aware of and meet physical activity recommendations. The physical activity guidelines (developed based on the World Health Organization’s Global Strategy on Diet, Physical Activity, and Health) [2] may also need to be adapted to be more culturally appropriate; the example exercises include ballroom dancing and swimming, which were not mentioned as activities the participants currently engage in (except expressing that they did not know how to swim).

Participants reported a preference for an in-person physical activity program, and they were especially interested in one based on group dynamics (i.e., designed to increase group cohesiveness [32, 41]). While one of the focus group questions asked participants what type of group-based activities would be helpful (with probes for setting group goals, social interactions, and supporting each other), participants provided unsolicited meaning units throughout the focus group related to other components of group dynamics. For example, participants mentioned group norms *(“I would love to see that everybody is taking part. Every member of the group is taking part in all the exercises”)* and group distinctiveness *(“So you just go there and get a shirt, which also helped to easily identify the Church membership exercise group wherever they went”)*. This preference for group-based activities is similar to the findings on preferences of women living in Accra and is supported in the literature, as group-dynamics physical activity programs have been shown to result in higher levels of adherence than individual programs, and are especially effective in older adults [32, 41]. A group-based physical activity program could be supplemented by other delivery methods (e.g., DVD, email, or WhatsApp), but the cost of internet access and gaining trust as a credible information source would need to be considered.

While the interviewees shared positive perceptions of physical activity, there are also barriers to overcome to help older adults achieve physical activity recommendations. One barrier related to program delivery was a lack of equipment; in particular, participants mentioned that they do not have access to weight lifting equipment. The physical activity program may need to include an evidence-based strength training routine using only body weight exercises or lower-cost equipment such as resistance bands.

Participants expressed both desire for a trained program leader and the ability to become leaders themselves once they had gone through the program. This indicates that a train-the-trainer model may work well as program participants can receive expert instruction to increase their confidence in becoming leaders. This model has been used successfully with community health workers and is commonly used in Extension physical activity programs [13, 30]. It can also decrease the cost required to sustain the program.

Finally, a “Keep Fit” club was mentioned by several participants as a current physical activity program in place at churches *(“So please, know that we have just formed a Keep Fit club in the church. We will meet at least once a month.”)*. Participants also mentioned intentions to start a Keep Fit club at the conclusion of the focus groups. Given the interest in this existing program, it may be possible to work with Keep Fit clubs to learn about current components of the program and suggest culturally appropriate adaptations to provide a structured program with evidence-based benefits for older adults’ health. For example, clubs that meet monthly could be encouraged to both add additional meetings and promote physical activities outside of the classes to help older adults meet physical activity recommendations. In addition to encouraging health care providers to recommend physical activity to their patients, a referral system could be set up (e.g., at diabetes clinics) to refer patients to the physical activity program.

### Limitations

The original research design was to conduct two focus group in each of the three cities; however, one focus group in Accra was cancelled due to scheduling difficulties. One additional church was added in Koforidua (where the research team was based) due to participant interest, and due to large numbers of interested participants at both Koforidua churches, participants were split into smaller groups. A focus group at the Koforidua diabetes clinic was added due to local interest in the role of physical activity in diabetes management; these participants were also split into two smaller groups. These changes resulted in the majority of the focus groups taking place in Koforidua. While this meant that the sampling was not representative of the three selected regions, we believe that the results provide useful data to complement the existing research conducted in Accra (1,594,000), which is much larger than Koforidua (population 122,300).

Even with splitting the large numbers of interested participants into smaller groups, the number of participants in six of the focus groups was larger than the recommended six to ten participants [25, 26]. This made the discussions more difficult to facilitate. While the co-moderator captured field notes (e.g., participants’ body language such as nodding in agreement), the large number of participants within each focus group made it difficult to include these field notes in our analysis. For these reasons, frequencies of subthemes and categories were reported across focus groups rather than only by the number of individuals who provided the specific meaning units. Additionally, we were not able to match the meaning units with the participant who provided them. Providing participant information with each quote in the text (e.g., age, gender, and physical activity level) would have provided helpful context. There were also language barriers with some of the focus group questions that required clarifications for participants.

As for the questionnaire, the GPAQ was been developed for face-to-face interviews conducted by trained interviewers; however, we included it as a questionnaire item. Some participants read and completed the questionnaire on their own, while others had the survey read to them by volunteer assistants. We did not use the adapted self-administered version of the GPAQ; while this version may have been more appropriate for our questionnaire, it does not contain introductory statements (e.g., “Next I am going to ask you about the time you spend doing different types of physical activity in a typical week”) which were likely helpful when our assistants read the questionnaire to participants with limited English reading skills. Also, the questionnaire did not fully assess whether participants are meeting Ministry of Health physical activity guidelines, as the GPAQ does not include strength training items. While qualitative results show that participants need clarification on what counts as strength training, some participants did mention engaging in strength training (e.g., press-ups and lifting weight), so we do not know if participants are achieving strength training recommendations. In addition, the questionnaire asked participants to self-report their weight status. Weighing and measuring participants at the focus groups would have been a more valid alternative; however, this was not a primary aim of our study and we chose a less intrusive measurement that may have been more culturally sensitive.

Finally, we recognize that the presence of an American researcher and the compensation for participating in the focus groups may have contributed to the high focus group participation rates. Participation rates may not be as high for a community-based program sustained after the research team leaves.

## Conclusion

This study addresses physical activity perceptions and preferences of male and female older adults in urban areas of Ghana in a novel and robust way. The results presented here suggest that a group-based physical activity program encouraged by health care providers and delivered at churches through a train-the-trainer model would be well received by older adults. In addition, education on physical activity recommendations and types is needed along with better dissemination of the Ministry of Health physical activity guidelines. Understanding older adults’ perceptions of physical activity and desired program characteristics can lead to development of a physical activity program that is acceptable, appropriate, and feasible for the target population.

## Additional files

Additional file 1: Focus group script. (DOCX 15 kb)

Additional file 2: Focus group questionnaire. (DOCX 17 kb)

## Abbreviations

BMI: Body mass index
FG: Focus group
GPAQ: Global physical activity questionnaire
IPAQ: International physical activity questionnaire
MET: Metabolic equivalent task
MU: Meaning unit
NIFA: National institute of food and agriculture
SAGE: Study on global AGEing and adult health
