# Supplementary material for: Older Ghanaian adults’ perceptions of physical activity: an exploratory, mixed methods study
Source: BMC Geriatr. 2019 Mar 15;19:85. doi: 10.1186/s12877-019-1095-1 (PMC6419803; doi:10.1186/s12877-019-1095-1)
Supplement: Supplementary file 1 — Focus group script. (DOCX 15 kb) [file 12877_2019_1095_MOESM1_ESM.docx]

Additional file 1: Focus group script

**Older Ghanaian Adults’ Perceptions of Physical Activity**

**Focus Group Script**

Thank you for joining us today to participate in the physical activity focus group. A focus group is an interview with multiple persons at once in order to gain a rich understanding of a particular topic. Today we want to discuss your perception of physical activity as well as opinions on the best ways to assist older adults with being more active. There will be approximately 64 participants from Accra, Cape Coast, and Koforidua taking part in focus groups. The length of time you can expect to be in this focus group is 90 minutes.

I am _______, and I am part of the research team. We will start by talking about your role as a participant in this study, obtaining informed consent, and completing a brief questionnaire. Then, I will start the focus group.

We welcome your responses either in Twi or English. For this reason, Laura, who is here to assist me, will be stopping us after each section of the interview to ensure we got to all the questions.

First I will read the consent form, allow time for any related questions, and collect signed consent for those of you who wish to continue with this focus group interview.

*(Read consent form, collect signed consent forms. Participants complete questionnaire. Resume [~15 minutes]).*

There are no right or wrong answers, so please share your experience and thoughts as we continue. Again, Laura will keep track of time. When she pauses us, it’s to check that we have covered all the topics of interest.

I will begin recording now.

Please state the number assigned to you in your packet.

*(Allow participants to read their assigned participant number to both test for volume and ‘record’ voice recognition.)*

Thank you for joining.

**Our first few questions are related to your perceptions of physical activity (~30 minutes).**

*Attitude (not read)*

- Please tell me about the positive things that may happen if you exercise.
- Please tell me about the negative things that may happen if you exercise.

*Subjective Norms (not read)*

- The ministry of health recommends engaging in 150 minutes of moderate-intensity aerobic activity (30 minutes most days of the week) and two sessions of muscle-strengthening activities (targeting major muscle groups) per week. Tell me how you feel about this recommendation.
- What would it take for someone to convince you that it is important to do aerobic activity for 30 minutes most days of the week?
  - Probes: who, how, why, health care provider
- What would it take for someone to convince you that it is important to do muscle-strengthening activities two days per week?
  - Probes: who, how, why, health care provider

*Behavioral Intention (not read)*

- Please describe your intentions to do aerobic activity for 30 minutes most days of the week.
- Please describe your intentions to do muscle-strengthening activities two days per week?

*Implementation Intention (not read)*

- If you plan to be do aerobic activity for 30 minutes most days of the week, how would you do so?
  - Probes: when, where, how
- If you plan to do muscle-strengthening activities two days per week, how would you do so?
  - Probes: when, where, how
- If you already meet these recommendations, how will you maintain these behaviors?

*Perceived Behavioral Control (not read)*

- What would make it easy to meet physical activity recommendations?
- What would make it hard to meet physical activity recommendations?
- What would you need to help you meet physical activity recommendations?

**The next few questions are related to developing a physical activity program for older adults in Ghana (~30 minutes).**

We are working to develop an appropriate physical activity program for older adults in Ghana.

- How do you feel about an in-person physical activity program?
  - Probe: What do you want the class to focus on? Have you ever attended a similar class? If so, what was your experience?
- How often should the class meet?
- How long should the class be?
- Where would it be convenient for the classes to meet?
  - Town:
  - Building type:
  - Church:
- What would you do in this class?
- Should separate classes be offered for men and women, or should they participate together?
- Think about who should teach this class. What type of person would be most helpful?
  - Probe: how old, male or female, faith-based, what would s/he do that would be most helpful?
- What program characteristics would you prefer to see in a physical activity program?
  - Probes: tracking, in a group, one-on-one, social support, feedback on goals, diaries.
- What type of group based activities would be helpful?
  - Probes: setting group goals, social interactions, supporting each other
- What should be included to teach you how to eat when participating in a physical activity program?
- How do you feel about health promotional information delivered via:
  - DVD
  - Text
  - Online
  - Email
  - In-person
- What types of incentives would motivate people to get involved in the program?
  - Probe: What type of incentives would help them stay involved?
- What do you think could make it hard for older adults to participate in the program?
  - Probe: What would help them overcome these barriers?
- After going through a program like this, do you think you could teach it yourself?
  - Probe: What would it take for you to feel comfortable teaching the program?

*Wrap-Up (~15 minutes)*

Is there anything else you would like to share with the research team at this time?

**I am going to stop recording now.**

Thank you for your participation in this focus group. Members of the research team will transcribe these sessions verbatim (or, word for word). We will then interpret the findings in order to develop a program for older adults in Ghana. If at any time during this process you wish to retract all or part of your statements, you may do so. You have our contact information on your consent form documents.
