# Supplementary material for: Older Ghanaian adults’ perceptions of physical activity: an exploratory, mixed methods study
Source: BMC Geriatr. 2019 Mar 15;19:85. doi: 10.1186/s12877-019-1095-1 (PMC6419803; doi:10.1186/s12877-019-1095-1)
Supplement: Supplementary file 2 — Focus group questionnaire. (DOCX 17 kb) [file 12877_2019_1095_MOESM2_ESM.docx]

Additional file 2: Focus group questionnaire

**Older Ghanaian Adults’ Perceptions of Physical Activity Focus Group**

Questionnaire

PART A: Tell us about yourself.

**1)** **Age** ________

**2)**  **Sex**

🞏1 Male

🞏2 Female

🞏3 Prefer not to answer

**3)**  **My weight status is classified as:**

🞏1 Underweight

🞏2 Normal weight

🞏3 Overweight

🞏4 Obese

🞏5 Don’t know

**4) Please indicate which of the following best describes you (choose all that apply):**

🞏1 Akan

🞏2 Ewe

🞏3 Ga-Adangbe

🞏4 Gruma

🞏5 Grusi

🞏6 Guan

🞏7 Mande-Busanga

🞏8 Mole-Dagbon

🞏9 Other (please specify) ___________________________

**5) Please mark the highest level of school that you have completed.**

🞏1 No formal education

🞏2 Primary school

🞏3 Secondary high school

🞏4 High school completed

🞏5 College

**6) What is your employment status?**

| 🞏1 Currently working | 🞏4 A homemaker |
| --- | --- |
| 🞏2 Never worked | 🞏5 Retired |
| 🞏3 Not currently working | 🞏6 Disabled/unable to work |

**7) What is your marital status?**

🞏1 Single 🞏2 Married 🞏3 Widowed

🞏4 Separated 🞏5 Divorced 🞏6 Living with partner

**8) How long did it take you to get to the church today?**

🞏1 <10 minutes

🞏2 11-20 minutes

🞏3 21-40 minutes

🞏4 > 40 minutes

**9) How did you travel to church (e.g., walk, bus, car, someone drove you)?**

**_______________________________________________________________________**

**10) Where do you live?**

🞏1 Accra

🞏2 Cape Coast

🞏3 Koforidua

🞏4 Other (please specify) ___________________________

**Part B. Health**

**1) In general, compared to other persons your age, how would you rate your health?**

🞏1 Extremely healthy

🞏2 Somewhat healthy

🞏3 Not healthy

🞏4 Very unhealthy

🞏5 Don’t know

**2) How confident are you that you can engage in moderate physical activities (e.g., not exhausting, light perspiration) for 30 minutes for 5 or more days per week?**

🞏1 Not at all 🞏2 Somewhat 🞏3 Moderately 🞏4Very 🞏5 Completely

**Next I am going to ask you about the time you spend doing different types of physical activity in a typical week. Please answer these questions even if you do not consider yourself to be a physically active person.**

**Think first about the time you spend doing work. Think of work as the things that you have to do such as paid or unpaid work, study/training, household chores, harvesting food/crops, fishing or hunting for food, seeking employment. In answering the following questions 'vigorous-intensity activities' are activities that require hard physical effort and cause large increases in breathing or heart rate, 'moderate-intensity activities' are activities that require moderate physical effort and cause small increases in breathing or heart rate.**

***Work***

**3) Does your work involve vigorous-intensity activity that causes large increases in breathing or heart rate like carrying or lifting heavy loads, digging, or construction work for at least 10 minutes continuously?**

🞏1 Yes

🞏2 No 🡪 If no, go to **question 6**

**4) In a typical week, on how many days do you do vigorous intensity activities as part of your work?**

___ Days a week

**5) How much time do you spend doing vigorous-intensity activities at work on a typical day?**

**___**Hours: ___ minutes

**6) Does your work involve moderate-intensity activity that causes small increases in breathing or heart rate such as brisk walking or carrying light loads for at least 10 minutes continuously?**

🞏1 Yes

🞏2 No 🡪 If no, go to **question 9**

**7) In a typical week, on how many days do you do moderate intensity activities as part of your work?**

___ Days a week

**8) How much time do you spend doing moderate-intensity activities at work on a typical day?**

**___**Hours: ___ minutes

***Travel to and from places***

**The next questions exclude the physical activities at work that you have already mentioned. Now I would like to ask you about the usual way you travel to and from places. For example to work, for shopping, to market, to place of worship.**

**9) Do you walk or use a bicycle (pedal cycle) for at least 10 minutes continuously to get to and from places?**

🞏1 Yes

🞏2 No 🡪 If no, go to **question 12**

**10) In a typical week, on how many days do you walk or bicycle for at least 10 minutes continuously to get to and from places?**

___ Days a week

**11) How much time do you spend walking or bicycling for travel on a typical day?**

**___**Hours: ___ minutes

***Recreational activities***

**The next questions exclude the work and transport activities that you have already mentioned. Now I would like to ask you about sports, fitness and recreational activities (leisure).**

**12) Do you do any vigorous-intensity sports, fitness or recreational (leisure) activities that cause large increases in breathing or heart rate like running or football for at least 10 minutes continuously?**

🞏1 Yes

🞏2 No 🡪 If no, go to **question 15**

**13) In a typical week, on how many days do you do vigorous intensity sports, fitness or recreational (leisure) activities?**

___ Days a week

**14) How much time do you spend doing vigorous-intensity sports, fitness or recreational activities on a typical day?**

**___**Hours: ___ minutes

**15) Do you do any moderate-intensity sports, fitness or recreational (leisure) activities that cause a small increase in breathing or heart rate such as brisk walking or volleyball for at least 10 minutes continuously?**

🞏1 Yes

🞏2 No 🡪 If no, go to **question 18**

**16) In a typical week, on how many days do you do moderate intensity sports, fitness or recreational (leisure) activities?**

___ Days a week

**17) How much time do you spend doing moderate-intensity sports, fitness or recreational (leisure) activities on a typical day?**

**___**Hours: ___ minutes

***Sedentary behavior***

**The following question is about sitting or reclining at work, at home, getting to and from places, or with friends including time spent sitting at a desk, sitting with friends, traveling in car, bus, train, reading, playing cards or watching television, but do not include time spent sleeping.**

**18) How much time do you usually spend sitting or reclining on a typical day?**

**___**Hours: ___ minutes

**19) What are the physical activity recommendations for older adults?**

🞏1 30 minutes of moderate intensity physical activity 5 days a week

🞏2 20 minutes of moderate intensity physical activity 3 times per week

🞏3 60 minutes of moderate intensity physical activity most days of the week

🞏4 Unsure

**20) The amount of physical activity I engage in is:**

🞏1 Less than the recommended amount of physical activity

🞏2 Meeting physical activity recommendations

🞏3 More than the physical activity recommendations

🞏4 I do not engage in physical activity

🞏5 Unsure
